# Beyond Soft Hands: Efficient Grasping With Non-Anthropomorphic Soft Grippers

**DOI:** 10.3389/frobt.2021.632006

**Published:** 2021-07-07

**Authors:** Yufei Hao, Yon Visell

**Affiliations:** ^1^ Soft Transducers Laboratory, École Polytechnique Fédérale de Lausanne, Neuchâtel, Switzerland; ^2^ Media Arts and Technology Program, Department of Electrical and Computer Engineering, Department of Mechanical Engineering, University of California, Santa Barbara, Santa Barbara, CA, United States

**Keywords:** soft robotics, robot hands, non-anthropomorphic grippers, soft grippers, adhesion, morphological adaptation

## Abstract

Grasping and manipulation are challenging tasks that are nonetheless critical for many robotic systems and applications. A century ago, robots were conceived as humanoid automata. While conceptual at the time, this viewpoint remains influential today. Many robotic grippers have been inspired by the dexterity and functionality of the prehensile human hand. However, multi-fingered grippers that emulate the hand often integrate many kinematic degrees-of-freedom, and thus complex mechanisms, which must be controlled in order to grasp and manipulate objects. Soft fingers can facilitate grasping through intrinsic compliance, enabling them to conform to diverse objects. However, as with conventional fingered grippers, grasping *via* soft fingers involves challenges in perception, computation, and control, because fingers must be placed so as to achieve force closure, which depends on the shape and pose of the object. Emerging soft robotics research on non-anthropomorphic grippers has yielded new techniques that can circumvent fundamental challenges associated with grasping *via* fingered grippers. Common to many non-anthropomorphic soft grippers are mechanisms for morphological deformation or adhesion that simplify the grasping of diverse objects in different poses, without detailed knowledge of the object geometry. These advantages may allow robots to be used in challenging applications, such as logistics or rapid manufacturing, with lower cost and complexity. In this perspective, we examine challenges associated with grasping *via* anthropomorphic grippers. We describe emerging soft, non-anthropomorphic grasping methods, and how they may reduce grasping complexities. We conclude by proposing several research directions that could expand the capabilities of robotic systems utilizing non-anthropomorphic grippers.

## 1 Introduction

Grasping, which is an indispensable operation for many robots, has been the subject of intense research over several decades. To operate effectively in many application scenarios, particularly in uncertain or unstructured environments, a robotic gripper should have the ability to grasp objects of various sizes and shapes in a variety of poses. These objectives have advanced the development of an array of anthropomorphic grippers (Melchiorri and Kaneko, 2008). Such grippers have been inspired by the multiple degrees of freedom and multi-modal perception of human hands. Many are thus equipped with multiple fingers and multiple sensors for capturing the positions of the digits and forces they apply. These resources can enable such grippers to grasp or manipulate objects by actively changing their configurations and applying sufficient forces to achieve grasping objectives (Pollard, 2004; Paolini et al., 2014; Ficuciello et al., 2019). While such grippers are attractive for their potential for accomplishing diverse grasping tasks, achieving such tasks involves complex control challenges, because the locations of fingers, and the forces they produce, must be regulated in order to achieve feasible grasps by positioning finger contacts and forces in feasible grasping configurations. Solving such problems often requires the application of real-time sensing and computer vision methods for inferring the poses, shapes, and features of grasped objects, and online planning computations for determining grasp trajectories. In conventional rigid robotic systems, these operations must be very precise in order to avoid damaging grasped objects. Underactuated gripper designs, particularly those that employ compliant fingers, can improve robustness and reduce control complexity in grasping, because their compliance allows them to passively adapt to the shape of grasped objects (Catalano et al., 2014; Odhner et al., 2014; Della Santina et al., 2015; Yoon and Choi, 2021). Nonetheless, grasping with such grippers often requires that the finger positions be adjusted to account for large variations in the three-dimensional geometries and poses of different objects. In addition, such designs often employ rigid contact surfaces that make it challenging to safely handle soft objects.

More recently, it has been observed that soft fingered grippers, which involve low elastic modulus materials, can reduce the demands on control and planning by virtue of the ability of compliant adaptation to the shape of grasped objects. These attributes can be understood as furnishing forms of morphological computation that can aid grasping (Shintake et al., 2018), especially where fragile objects are concerned. For the purpose of this article, we refer to these soft grippers as “anthropomorphic” by virtue of their discrete, compliant, finger-like elements that are each brought into contact with grasped objects. The compliance of these elements often allows such soft, fingered grippers to perform grasping *via* simplified bending-mode actuation of the fingers (Deimel and Brock, 2013; Mosadegh et al., 2014; Yuk et al., 2017; Abdullah et al., 2018). However, it is challenging to grasp objects of widely varied properties (e.g., sizes, shapes, and masses) with such grippers. This has led many researchers to investigate soft, fingered grippers of increasing complexity in order to improve the dexterity of the gripper and diversity of grasping tasks that can be accomplished (Hao et al., 2017; Yang et al., 2017; Hao et al., 2018; Zhou et al., 2018).

Grasping also plays a critical role in behaviors of many biological organisms, including predation and feeding, that are instrumental to survival. Many animals use grasping methods and mechanisms that are markedly different from those associated with the human hand. A noteworthy example is the gecko, which possesses feet with nanostructured setae that allow it to easily climb up vertical surfaces *via* adhesion. This remarkable ability has informed the engineering of devices including robotic grippers that apply similar principles of adhesion, enabling them to grasp objects with minimal gripping forces (Hawkes et al., 2017; Song et al., 2017). Another interesting reptilian example is the chameleon tongue. In chameleon predation, the capture of prey is facilitated by tongue specializations that allow the tip to adhere to and envelop prey. Several soft robotic grippers have been designed to use similar principles of enveloping, such as the FESTO adaptive shape gripper, which is able to grasp objects of various shapes by enveloping, among several other examples (Brown et al., 2010; Shintake et al., 2016; Li et al., 2019; Hao et al., 2020a). Biological systems such as these, and the robotic devices they have inspired, illustrate how non-anthropomorphic grippers can circumvent complexities that arise in fingered grasping, such as those of identifying feasible contact points and of controlling the motions and forces produced by multiple fingers.

In this perspective, we first identify fundamental challenges that arise in the use of human-hand-inspired, “anthropomorphic” grippers, which often require complex systems and methods be applied in order to meet contemporary requirements for robotic grasping. We then discuss how emerging, non-anthropomorphic grasping mechanisms can reduce the complexity of grasping tasks, and consequently simplify the systems involved. We discuss several paradigms for non-anthropomorphic grasping that are reflected in recent research findings, as we highlight through selected examples from the literature. We then conclude by synthesizing prospects for non-anthropomorphic grippers in robotics, and discuss interesting challenges that remain to be solved for these soft grippers to attain their potential.

## 2 Diverse Grasping Requirements in Robotics: Motivation for New Design Paradigms

Traditional industrial processes in manufacturing and other areas have required grippers, processes, and materials to be configured in highly constrained ways in order to insure that standardized objects, such as automotive parts or food items, can be consistently handled. Such processes have often also required that the handled objects be presented in known poses and configurations. Today, there is increasing demand from areas of industry, including logistics, on-demand manufacturing, and service robotics, for robotic grippers that can grasp objects that vary greatly in size, shape, position, orientation, and environmental clutter without altering the end effector on a case-by-case basis. Such requirements can, in principle be met through the use of fingered grippers. However, achieving such aims with fingered grippers often requires significant sensing and computational resources in order to sense the object and environment state, and determine and achieve appropriate contact points and forces so as to achieve form and force closure for objects of varying size and shape (Pollard, 2004; Paolini et al., 2014). Several researchers have developed creative gripper designs that are better capable of addressing these challenges. Examples include grippers possessing fingers whose mounting configuration can be altered on-demand (Odhner et al., 2014), and grippers that possess fingers (Hao et al., 2020b) or palms (Subramaniam et al., 2020) that can be altered in dimensions in order to grasp objects of varying size or configuration.

At the same time, there is increasing demand for robotic systems that are able to handle soft or fragile objects, such as food, glass, electronic devices, or deformable bags, while still being able to handle heavy objects. These demands often involve opposite objectives to those arising from object diversity (as detailed above). On one side, soft and compliant grippers are very attractive for use in handling fragile objects without damaging them. On the other, higher levels of stiffness are beneficial when substantial loads must be handled. To meet these competing requirements, several researchers have proposed soft robotic grippers whose stiffness may be varied, such as by effecting active changes in composite material structures (Wei et al., 2016; Zheng et al., 2018). Such grasping devices are complex to fabricate and control, and have not yet been found capable of handling objects of widely varying properties.

Upon closer examination of the human hand and its modes of operation and behaviors (Xiong et al., 2016), it becomes apparent that the hand’s generalized grasping abilities are supported through multiple structural and functional specializations, including fingers with several degrees of freedom, a reconfigurable palm that allows the grasping workspace to be altered, and a serendipitous combination of rigid structural elements (bones) with an elaborate network of soft and connective tissues. Equally important are the sensory organs of vision and of touch—including the skin [itself an exquisitely refined sensory organ (Biswas and Visell, 2019)], along with the brain’s impressive capacities for perception, cognition, and motor control. These capacities are all instrumental to the remarkable prehensile abilities of the human hand. Together, they allow us to gently handle small or delicate objects, such as uncooked eggs, while also manipulating large loads, like heavy suitcases. It would be prohibitively complex and costly to design a robotic hand that mimics the many intricate aspects of the human hand that enable such activities. Thus, fundamentally different robotic gripper designs may be needed in order to meet current and future requirements for practical robotic grasping in industry and other areas.

## 3 Emerging Non-anthropomorphic Grasping Paradigms

For the reasons explained above, it is challenging for anthropomorphic, soft fingered grippers to grasp objects of various sizes, shapes, stiffnesses, and other attributes. The challenges involved can be traced to several fundamental limitations affecting such grippers. One arises from the impracticality of using humanoid grippers to emulate the human hand’s ability to change shape in order to adapt to the target or desired grip. Another concerns the mode in which the fingers are commonly actuated, *via* (typically) a single bending movement. Such motions introduce limitations that can prevent effective grasping in constrained or cluttered settings due to the workspace occupied by the finger motion paths (from open to closed), and can prevent effective grasping of objects in asymmetric orientations relative to the gripper.

In contrast, emerging non-anthropomorphic gripping methods in soft robotics can more readily accommodate variations in object size, shape, and pose, by morphologically adapting to the object shape. Such abilities can also allow these kinds of grippers to be actuated using simple methods involving fewer actuated degrees-of-freedom. In contrast to anthropomorphic grippers, which often require multi-degree-of-freedom actuation, many non-anthropomorphic grippers require the actuation of few degrees-of-freedom for grasping, which is possible because their deformability ensures that their shape is dominated by that of a contacted object. Consequently, such grippers can be more easily operated, without need for complicated control methodologies, camera-based geometry inference, or multi-sensor feedback.

While an array of non-anthropomorphic grippers have been recently investigated, they remain substantially outnumbered by anthropomorphic robotic grippers. Comparatively few non-anthropomorphic gripper designs have reached levels of functionality needed for real-world applications. Nonetheless, several types of non-anthropomorphic soft gripper have been described in the literature to date, some of which mirror methods of gripping that are present in nature. Prominent categories include grippers based on non-anthropomorphic fingered limbs, based on enveloping, wrapping, adhesion, and suction. For the purpose of this article, we exclude grasping methods that could involve damage to the grasped object. Such methods include piercing or pinching *via* a sharp structure such as a sharp rod or avian beak. We also omit a discussion of fingered grippers with more or fewer than five digits, because most of the considerations detailed above also apply to such grippers.

### 3.1 Enveloping and Wrapping

One emerging category of soft robotic gripper design is based on the idea of grasping objects *via* a contiguous deformable surface that encloses the grasped object without recourse to finger-like protuberances. Such approaches have often been motivated with the objective of grasping and handling objects of diverse shapes using simple actuation methods. Methods that we refer to as *enveloping* involve partially enclosing an object within a three-dimensional region of a soft surface. In wrapping methods, an extended structure, similar to a tendril or tentacle, is wrapped around the grasped object.

**FIGURE 1:**
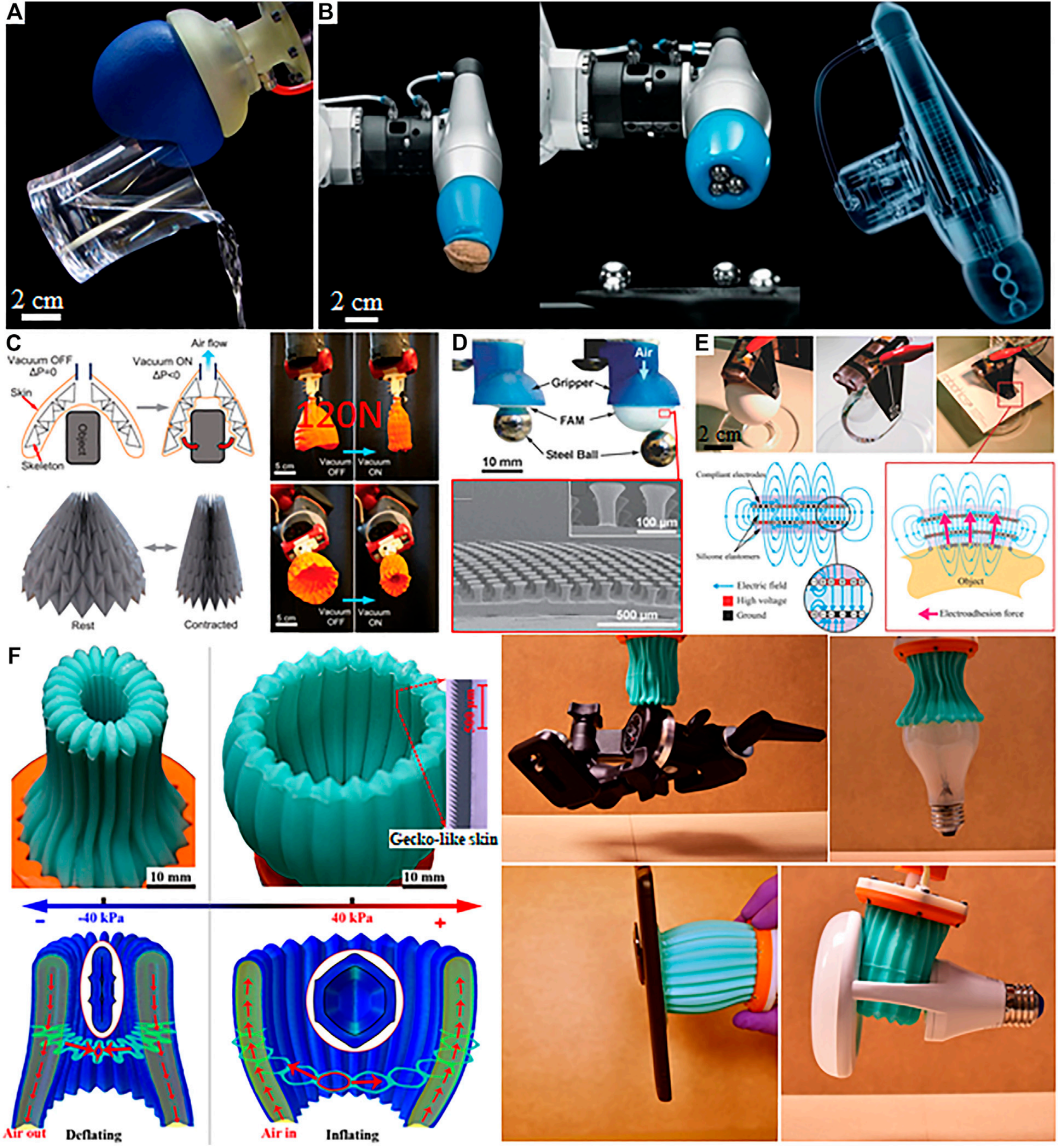
Representative non-anthropomorphic grippers: **(A)** jamming gripper (Brown et al., 2010), **(B)** adaptive shape gripper, **(C)** origami “magic ball” gripper (Li et al., 2019), **(D)** gecko adhesion-based gripper (Song and Sitti, 2014), **(E)** electroadhesion based gripper (Shintake et al., 2018), **(F)** multimodal enveloping gripper (Hao et al., 2020a).

One efficient design for grasping *via* partial envelopment is the universal jamming gripper of Brown et al. (2010). The gripper structure consists of a simple elastic membrane filled with a fine granular medium—ground coffee in the version described in the original work. The granular medium is initially in a loose state within the balloon-like membrane. This gripper structure deforms in contact with an object, forming a large contact surface. Vacuum is subsequently applied to the interior of the gripper, causing the granular medium to jam and become rigid. This allows the gripper to produce substantial lifting forces (Figure 1A). The ability of this gripper to conform to diverse shapes allows it to grasp a wide variety of objects.

Another example is the FESTO adaptive shape gripper mentioned in the introduction. This gripper consists of a double-acting soft cylinder comprising two chambers. One chamber is filled with compressed air while the other is filled with water. The water-filled chamber is a silicone cap, inspired by the tongue of a chameleon (Figure 1B). During grasping, the gripper first contacts an object *via* the silicone cap. When the top pressurized chamber is vented, the piston moves upward and the silicone cap contracts. As the gripper further intrudes upon the object, the silicone cap encloses the object at the contact surface, thereby forming a firm fit to the contact surface. This allows the gripper to grasp objects of diverse shapes. Large grasping forces can be sustained with little energy consumption due to the high static friction coefficient of the contacting material.

An alternative approach is used in the magic ball gripper of Li et al. (2019). This device consists of a folded, origami-like structure with an aperture at one end, which is constructed from an elastic membrane. Applying fluidic vacuum to the gripper causes the aperture to contract from a larger to smaller shape in a manner similar to a closing flower (Figure 1C). The resulting contraction allows the gripper to enclose any object that is able to fit within the opening.

A variety of soft robotic grippers or other devices have been designed to wrap around objects or structures. Popular sources of inspiration for such grippers are the octopus arm and the elephant trunk, which have inspired several groups to develop soft robotic manipulators that can wrap around objects in bioinspired fashion (McMahan et al., 2006; Calisti et al., 2011; Hoang et al., 2020).

### 3.2 Adhesion and Suction

Other methods for grasping *via* non-anthropomorphic soft grippers have been based on adhesion or suction produced by pressure differences. Both of these techniques have previously been applied in conventional robotic end effectors investigated in research laboratories or fielded in industrial applications.

In adhesion-based grippers, grasping forces are produced *via* the generation of enhanced adhesive friction forces, sometimes in ways that allow the adhesive forces to be dynamically controlled. Such mechanisms allow objects to be grasped and lifted with minimal normal force application. This can be advantageous when manipulating low-mass objects, or when grasping floating structures in microgravity during space exploration missions. One approach for adhesion-based grasping involves surface patterning, for which an analogy can be drawn to specialized skin on the volar forelimb of gecko lizards. As noted in the introduction, such gecko skin is patterned with setae whose multiscale structures greatly increase the true area of contact with touched surfaces, thus generating large adhesion *via* Van der Walls forces. The structure and mechanics of these setae ensure that shear loads are uniformly distributed, thereby further increasing the static friction coefficient that can be established between the gecko skin and touched surfaces. These principles have been applied for the engineering of adhesion-based devices, like the microfibrillar soft robotic gripper of Song and Sitti (2014). This gripper employs an elastic membrane patterned with microfibers attached to a fluidically actuated elastic body. In operation, the gripper body is inflated, brought into contact with the object, and then compressed upon it. Shear forces produced during lifting engage the microfibers allowing the object to be raised (Figure 1D).

Another method for gripping *via* adhesion, referred to as electroadhesion, involves the use of electrostatic forces. Electroadhesive grippers have found use in conventional automation systems for manipulating silicon wafers, paper, or textiles, and have more recently been investigated in soft robotics. Such grippers involve the application of large differences *V* in voltage (typically several kV) to conductive, interdigitated electrodes embedded in shallow layers of elastic contact pads. During operation, the paired electrodes form a capacitive structure whose capacitance *C* is increased when the pad is brought in contact with an object. When the gripper pad is electrified, a displacement of the object away from the pad by an amount *z* reduces the stored energy $U\left(z\right)=C\left(z\right)V^{2}/2$ in the capacitor and thus generates an attractive force F=dU/dz between the object and pad. Shintake et al. (2018) designed a soft, thin robotic gripper formed from conductive polymer layers, and demonstrated the ability of this gripper to grasp a variety of objects (Figure 1E).

Suction provides another method for gripping objects *via* adhesion without the need for fingers. Such methods have been widely used in industrial robotics. Suction methods involve the formation of a complete or partial seal with the object surface, and the production of a lower than atmospheric pressure region across the sealed region, thus yielding an attractive force proportional to the sealed surface area and to the pressure difference. In the presence of this adhesive force, the contacting surfaces bounding the sealed region produce shear forces that can improve grasping stability by preventing objects from translating in lateral directions, orthogonal to the direction of contact. In nature, and in everyday objects, soft suction cups provide means of attachment to objects. Inspired by the simplicity and ubiquity of these methods, several groups have investigated soft robotic systems that leverage suction for grasping. For example, among the octopus arm inspired soft manipulators reported in the literature, several have integrated suckers to aid object grasping (McMahan et al., 2006; Xie et al., 2020). In underwater environments, due to the denser fluid medium, a soft gripper can be augmented with suction to aid the handling or capture of nearby small objects (Stuart et al., 2018).

### 3.3 Multimodal Grasping

It can be advantageous to adopt a multimodal approach to robotic grasping, by combining different grasping methods with attributes suited to different grasping tasks or objects. Recently, we designed a non-fingered soft gripper that leverages several different gripping mechanisms: enveloping, adhesion, suction, and inflation. We demonstrated the ability of the same gripper to grasp a variety of objects in different ways (Hao et al., 2020a). The gripper body consists of an accordion-shaped elastic cylindrical shell with an aperture at one end. The elastic shell contains an array of parallel chambers linked to a common fluidic port. Inflating the chambers, through the application of positive pressure, causes the aperture to open, allowing objects to be swallowed within the interior. Deflating the gripper causes the aperture to close and conform to the object’s shape, enveloping the object (Figure 1F). A gecko-skin-like polymer layer on the interior walls of the gripper increases the lifting forces that can be produced, allowing large objects to be handled, while a wall buckling mechanism intrinsic to this design allows the gripper to manipulate even fragile objects without damage. We also demonstrated how this soft robotic end effector can lift flat objects, by contacting them to form a seal and inflating to produce a pressure differential. The gripper is also able to lift objects by inflating to fill an opening or handle on the object itself.

## 4 Discussion and Future Research Directions

Traditional approaches to robotic grasping often involve the use of anthropomorphic or other fingered grippers. As we highlight here, non-anthropomorphic soft grippers can circumvent challenges that arise in the application of fingered grippers to the grasping of diverse objects in varied conditions or unstructured environments.

We have surveyed an array of methods for grasping *via* soft, non-fingered grippers as reflected in recent soft robotics research. Despite their diversity, several common aspects of their designs stand out. Indeed, grasping methods embodied by many of these grippers can be characterized in terms of a few basic approaches that contrast with those used in fingered grippers. We have highlighted a few of the most common here, including those of enveloping, wrapping, adhesion, and suction. We have also described several soft robotic manipulators that use such approaches to implement multiple grasping strategies. Many non-fingered soft grippers share common characteristics and morphologies that are integral to their function. These include the presence of compliant or elastic surfaces that can conform to the shape and pose of diverse objects, the establishment of relatively large contact areas at which forces can be applied that are sufficient to ensure force balance and produce substantial shear forces, and the use of materials, structures, or active (fluidic or electrical) methods for enhancing frictional forces with objects. The same attributes can also allow such grippers to handle objects without applying excessive forces, thus reducing risks of damage to fragile objects.

What stands out as most remarkable about these systems is the simplicity with which many (and often multiple) grasping tasks can be accomplished using non-fingered grippers, despite the fact that they often possess far fewer actuated degrees of freedom than are present in anthropomorphic robotic hands. Several of the examples we have cited require just one actuated degree of freedom for their operation—for example, a single fluidic port. Many are also able to grasp objects without detailed knowledge about the object or scene geometry, and thus may be operated without three-dimensional geometry inference (e.g., from a computer vision system), or force sensors. Consequently, the instrumentation and computation requirements for operating such grippers can be significantly lower than are needed in fingered grippers, reducing costs and potentially increasing operation speeds. This suggests that non-anthropomorphic soft grippers could find practical application in many tasks in which robotic systems may not be cost effective today.

Although we have emphasized commonalities among non-anthropomorphic soft gripper designs, there exist several noteworthy differences among approaches that have been adopted to date, and the capabilities of the resulting systems. When compared with grippers based on adhesion and suction, those that employ enveloping or wrapping are arguably more versatile. Enveloping or wrapping grippers have the potential to grasp objects despite important variations in interfacial surface conditions (dry, wet, conductive or insulating). They can alter the size of grasping interfaces by adapting their structure or size, and can be designed to augment their load capacity through the use of stiffer materials that facilitate the variable, possibly higher, operating pressures. Grasping *via* wrapping can, however, introduce challenges associated with the fact that grasping is typically initiated from one side. Thus, such methods may be more suitable for grasping objects with larger length-to-width aspect ratios. For similar reasons, wrapping grippers may also be prone to reduced precision in gripper-object positioning when compared with enveloping grippers, due to the dynamically varying forces that are involved. While several of the enveloping and wrapping methods surveyed here have been demonstrated to be capable of grasping diverse objects, either approach can be applied in ways that yield large gripping forces that damage very fragile objects. In contrast, adhesion methods can be used in ways that provide greater safety, because of their reduced reliance on gripping forces. As mentioned, however, a disadvantage of adhesion methods is that the forces that are produced can vary greatly with interfacial surface conditions. For example, both gecko-like adhesion and electroadhesion are often unsuitable for use with wet or dirty interfacial surfaces. Electroadhesion-based grippers are also sensitive to the dielectric properties of the object and environment, which thus constrain their effective application. Shear-load limitations further constrain the effectiveness of adhesion based grippers in high load applications. In such cases, they may be combined with other grasping methods. The use of suction techniques is often limited to nearly flat, non-porous surfaces, and thus may need to be combined with other techniques if required to operate in diverse interfacial conditions.

Despite promising recent developments, several interesting challenges remain for research. One that can be readily identified in many studies concerns the sizes of objects that can be grasped. Several non-anthropomorphic soft grippers have been demonstrated to be capable of grasping a variety of objects in diverse conditions, but the range of sizes of objects that can grasped by a particular gripper is often limited. For example, the multimodal gripper of Hao et al. is able to grasp objects via features that span about half order of magnitude in dimensions (approximately 1–5 cm). Timely robotics applications in areas such as logistics and shipping involve the manipulation of objects, such as products in distribution warehouses, that vary tremendously in size. Thus, an interesting challenge for research is to design soft grippers that are able to handle larger or smaller objects. Another interesting Frontier concerns the application of adhesion-based grippers to the grasping of objects with highly curved or complex shapes, with which it is difficult to form seals or large contact surfaces with which adhesion forces can be produced. It is possible that this challenge may be met through the engineering of more complex gripper contact surfaces that integrate localized elements, akin to the tendrils of squid, that can fold around localized object features, or the smart films which can change shape to fully conform to objects. A further challenge arises from the demands for fast and precise pick-and-place manipulation in assembly line or other industry applications. With few exceptions, the speed and precision with which such tasks can be accomplished using soft, non-fingered grippers cannot yet match what is possible using conventional robotics, which arise in part because of the rigidity of conventional automata. To overcome these limitations, new methods for achieving higher precision and speed with soft grippers may be needed, perhaps through the use of composite material structures or variable stiffness methods.

Together, the promising recent discoveries that have been reported, and the compelling challenges that remain to be addressed, suggest that the engineering of soft, non-anthropomorphic grippers will continue to be among the most interesting areas of soft robotics research during the coming years.

## Data Availability

The original contributions presented in the study are included in the article/Supplementary Material, further inquiries can be directed to the corresponding author.
